# Draft Genome Sequence of Clostridium tyrobutyricum Strain DIVETGP, Isolated from Cow’s Milk for Grana Padano Production

**DOI:** 10.1128/genomeA.00213-15

**Published:** 2015-03-26

**Authors:** Alessio Soggiu, Cristian Piras, Stefano Gaiarsa, Emøke Bendixen, Frank Panitz, Christian Bendixen, Davide Sassera, Milena Brasca, Luigi Bonizzi, Paola Roncada

**Affiliations:** a Dipartimento di Scienze Veterinarie e Sanità pubblica (DIVET), Università degli Studi di Milano, Milano, Italy; b Microbiology and Virology Unit, Fondazione IRCCS Policlinico San Matteo, Pavia, Italy; c Department of Molecular Biology and Genetics, Aarhus University, Aarhus, Denmark; d Dipartimento di Biologia e Biotecnologie, Università degli studi di Pavia, Pavia, Italy; e Istituto di Scienze delle Produzioni Alimentari, CNR, Milano, Italy; f Istituto Sperimentale Italiano L. Spallanzani, Milano, Italy

## Abstract

We announce the draft genome sequence of Clostridium tyrobutyricum strain DIVETGP. This strain was isolated from cow’s milk used for Grana Padano cheese production. The genome was obtained using Illumina HiSeq technology and comprises 45 contigs for 3,018,999 bp, with a G+C content of 30.8%.

## GENOME ANNOUNCEMENT

Grana Padano is labeled as PDO (protected designation of origin) and is a raw-milk, long-ripened, and hard-cooked cheese. A typical problem with hard-cooked cheeses is the “late blowing” phenomenon, which is mainly due to the presence of high amounts of clostridia. Clostridium tyrobutyricum has been found to be the most frequently isolated species from late-blown Grana Padano cheese (1, 2). Clostridial endospores are naturally present in raw milk and grow during ripening, leading to butyric fermentation and associated gas production (3). Gas production in Grana Padano determines the formation of vacuoles in the structure of the cheese, which seriously depreciate its commercial value. This alteration of the product is accompanied by the onset of unwanted flavors and odors. In this work, a *de novo* shotgun sequencing of C. tyrobutyricum strain DIVETGP, isolated from Grana Padano raw milk used for cheese production, has been performed and the genomic sequence is reported only for the cheese-associated strain (4). Genomic DNA was fragmented (Covaris), and fragments in the range of 250 to 350 bp were selected for library preparation. Libraries were sequenced using an Illumina HiSeq2000 platform, generating about 638 million paired sequences with a fixed length of 100 bases. Reads were analyzed and quality checked using FastQC (5). A specifically designed Python script was used to filter low-quality data (i.e., terminal stretches with a quality score below 20). Genome assembly was performed using Velvet software (version 1.2.10) (6) after a process of parameter optimization with an in-house Perl script. The resulting assembly consists of 45 large contigs with an average coverage of 822× and an *N*_50_ of 168,596 bp for a total of 3,018,999 bp. Genome annotation was automatically performed on the RAST server (7) using Glimmer version 3 (8) for gene finding, obtaining 3,032 protein-coding genes. The availability of this draft genome sequence will enable a more in-depth comparative analysis of other *Clostridia* deriving from several dairy foods.

### Nucleotide sequence accession number.

The whole-genome shotgun project of C. tyrobutyricum DIVETGP has been deposited at DDBJ/EMBL/GenBank under the accession number CBXI000000000.
